# Suppressing molecular motions for enhanced room-temperature phosphorescence of metal-free organic materials

**DOI:** 10.1038/ncomms9947

**Published:** 2015-12-02

**Authors:** Min Sang Kwon, Youngchang Yu, Caleb Coburn, Andrew W. Phillips, Kyeongwoon Chung, Apoorv Shanker, Jaehun Jung, Gunho Kim, Kevin Pipe, Stephen R. Forrest, Ji Ho Youk, Johannes Gierschner, Jinsang Kim

**Affiliations:** 1 Department of Materials Science and Engineering, University of Michigan, Ann Arbor, Michigan 48109, USA; 2 Department of Electrical Engineering, University of Michigan, Ann Arbor, Michigan 48109, USA; 3 Department of Macromolecular Science and Engineering, University of Michigan, Ann Arbor, Michigan 48109, USA; 4 Department of Mechanical Engineering, University of Michigan, Ann Arbor, Michigan 48109, USA; 5 Department of Physics, University of Michigan, Ann Arbor, Michigan 48109, USA; 6 Department of Applied Organic Materials Engineering, Inha University, Incheon 402-751, Korea; 7 Madrid Institute for Advanced Studies—IMDEA Nanoscience, Calle Faraday 8, Ciudad Universitaria de Cantoblanco, Madrid 28049, Spain; 8 Department of Chemical Engineering, University of Michigan, Ann Arbor, Michigan 48109, USA; 9 Department of Chemistry, University of Michigan, Ann Arbor, Michigan 48109, USA; 10 Biointerfaces Institute, University of Michigan, Ann Arbor, Michigan 48109, USA

## Abstract

Metal-free organic phosphorescent materials are attractive alternatives to the predominantly used organometallic phosphors but are generally dimmer and are relatively rare, as, without heavy-metal atoms, spin–orbit coupling is less efficient and phosphorescence usually cannot compete with radiationless relaxation processes. Here we present a general design rule and a method to effectively reduce radiationless transitions and hence greatly enhance phosphorescence efficiency of metal-free organic materials in a variety of amorphous polymer matrices, based on the restriction of molecular motions in the proximity of embedded phosphors. Covalent cross-linking between phosphors and polymer matrices via Diels–Alder click chemistry is devised as a method. A sharp increase in phosphorescence quantum efficiency is observed in a variety of polymer matrices with this method, which is *ca*. two to five times higher than that of phosphor-doped polymer systems having no such covalent linkage.

## Abstract (web summary)

Phosphorescent materials made of purely organic components are typically less efficient than their organometallic counterparts. Here, the authors report a strategy to improve the phosphorescence efficiency of metal-free materials by reducing radiationless transitions by covalently linking into a polymer matrix.

## Graphical abstract

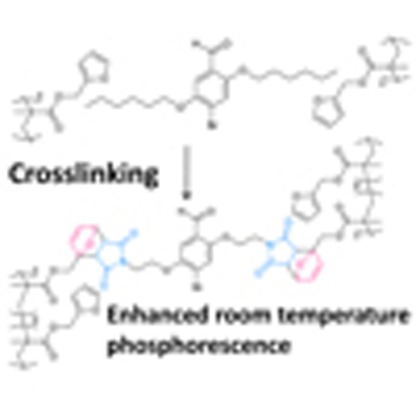

Metal-free room-temperature phosphorescent (RTP) materials are attractive alternatives to organometallic phosphors because of their low cost, earth-abundant constituent atoms and versatility of molecular design^1^. In particular, these materials have great potential for high-contrast bioimaging^2^, high-sensitivity optical sensing^3^,^4^,^5^,^6^,^7^, singlet fission materials^8^, unconventional reverse saturable absorption materials^9^ and advanced security imaging^10^. However, developing highly efficient metal-free RTP materials is very challenging, as, without heavy-metal atoms, both the rate of intersystem crossing (ISC) from the lowest excited singlet state (*S*_1_) to the triplet manifold (*T*_*n*_) and the rate of radiative deactivation from *T*_1_ to the ground state (*S*_0_) are typically slow, and therefore cannot compete with fluorescence, internal conversion (IC) and/or radiationless relaxation processes from *T*_1_ to *S*_0_ via ISC or quenching by the host matrix and/or oxygen, at ambient conditions (Fig. 1b).

ISC from *S*_1_ to *T*_*n*_ can be greatly promoted in special organic moieties through efficient spin–orbit coupling (SOC) by the effective mixing of the singlet and triplet states of different molecular orbital (MO) configurations (El-Sayed rule; for example, aromatic carbonyls and nitrogen heterocycles^11^,^12^,^13^) and by heavy halogens^14^, and/or through small singlet–triplet splitting energy (Δ*E*_ST_)^15^. However, in such compounds, the rate constant for phosphorescence is still small (*k*_P_∼10^2^ s^−1^), whereas in organometallic complexes, on the other hand, rate constants are as high as *k*_P_∼10^5^ s^−1^. Therefore, radiationless relaxation processes from *T*_1_ to *S*_0_ must be sufficiently suppressed to achieve bright RTP in metal-free organic phosphors.

A common method to enhance RTP in metal-free phosphors is to make them a crystal^10^,^14^,^15^,^16^,^17^,^18^,^19^ or to embed them into a carefully chosen matrix such as poly(vinyl alcohol)^5^,^20^,^21^, poly(methyl methacrylate) (PMMA)^4^,^22^,^23^,^24^, steroid analogue^6^,^9^,^25^, micelles^2^, the cavity of cyclodextrin^26^ or inorganic crystals^27^,^28^. Deuterium substitution has been another route developed to enhance RTP in metal-free phosphors, although it requires the rather synthetically difficult deuterium substitution process^6^,^9^,^25^. These studies have indicated that oxygen permeability, triplet energy and rigidity of the matrix, and deuterium substitution of the phosphor are all important contributors to the radiationless transitions. In particular, rigidity of the matrix is a very important parameter, which is also supported by the fact that polymer relaxation behaviour (for example, *α*-, *β*- and *γ*-relaxations) largely influences the phosphorescent emission properties of metal-free organic phosphors^4^,^22^. Although the influences of oxygen, the triplet energy of the host matrix and deuterium substitution of phosphors on the radiationless transitions have been well investigated^2^,^22^,^23^,^25^, the correlation between the radiationless relaxation pathways (for example, ISC from *T*_1_ to *S*_0_ and Dexter-type triplet energy transfer (ET) processes) and the rigidity of the environment is not fully understood, limiting the development of a general design principle to realize efficient RTP in a variety of polymer matrices. Moreover, the use of crystals and specific matrices can significantly reduce the practical utility due to difficult processing, undesired properties of matrices and mechanical fragility of those composites. Therefore, finding a new versatile method that enables the restriction of the radiationless relaxation pathways in a variety of amorphous polymer matrices, and that allows for a systematic study of their dependence on the rigidity of the environment, is highly desired, yet no such system has been reported.

Here we present a general design principle and a method to effectively reduce radiationless relaxation pathways and hence to achieve highly efficient RTP of metal-free organic materials in a variety of amorphous polymer matrices. Covalent cross-linking between phosphors and polymer matrices is devised as a method to suppress the molecular motions of embedded phosphors and polymer matrices. In fact, a cross-linking method has been used to improve thermal stability and solvent resistance of organometallic phosphor-based light-emitting devices^29^. We chose Diels–Alder click chemistry as the cross-linking method due to its modular and efficient nature, leading to convenient preparation and easy characterization of phosphor-doped polymer films. The resulting cross-linked phosphor-doped polymer system comprised of the designed diene-reactive phosphor (DA1) and ene-reactive polymer matrix showed highly efficient RTP with phosphorescence quantum efficiency up to *ca*. 28%, which is *ca*. 2–5 times higher than that of phosphor (Br6A)–polymer blend system having no such covalent linkages between the matrix and the phosphor (Fig. 1a). Detailed spectroscopic studies on both polymer blend systems reveal that restricting molecular motions in the proximity of phosphors (increasing the rigidity of their local environment) by cross-linking effectively reduces the collision frequency and hence the Dexter-type triplet ET processes and vibronic mixing between zero-order electronic states of *T*_1_ and *S*_0_ that directs the reduction of the rate of the ISC process (Fig. 1b). Moreover, by developing the DA1-copolymer systems with various monomers, we demonstrate that our method is a generally applicable strategy to achieve highly efficient metal-free RTP in a variety of polymer systems.

## Results

### Molecular design of DA1

**Figure 1:**
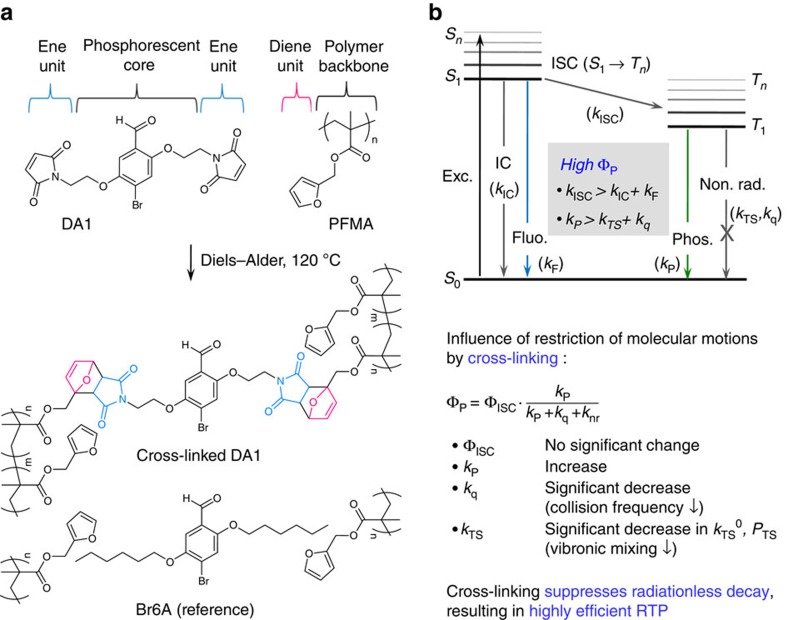
Chemical structures of designed phosphor and polymer and description of covalent cross-links strategy. (**a**) Chemical structures of the newly designed DA1 and PFMA are shown. Diels–Alder click reaction in DA1-doped PFMA blend film forms covalent bonds between DA1 and PFMA, resulting in greatly enhanced phosphorescence efficiency (*Φ*_P_) compared with Br6A-doped PFMA blend film having no covalent linkages. (**b**) A general Jablonski diagram of organic emitters is presented. General rules for efficient RTP emitters are shown in the grey box. The influence of cross-linking via the Diels–Alder reaction is described in the blue box. The restriction of motions of phosphors and matrices by cross-linking effectively reduces the collision frequency and hence the Dexter-type triplet ET processes (*k*_q_) and the vibronic mixing that directs the reduction of the rate of the ISC process from *T*_1_ to *S*_0_ (*k*_TS_).

We designed a clickable phosphor, DA1, through side-chain modification of Br6A, while maintaining the core structure of Br6A. For the side chains, we chose a maleimide moiety that enables the Diels–Alder click reaction with a furan group of a designed complementary polymer, poly(furfuryl methacrylate) (PFMA; Fig. 1a). The bromo/benzaldehyde design promotes ISC via SOC by the creation of an nπ*-type triplet (*T*_*n*_) state, due to the benzaldehyde moiety, as well as by the intra- and intermolecular heavy atom effect of bromine (however, this is moderate with respect to heavy metals)^16^. In fact, bromine promotes SOC by the direct participation in the relevant frontier *π*/π*-type MOs, providing efficient solid-state phosphorescence (*vide infra*)^13^,^14^. A full description of the synthetic approaches and ^1^H-NMR, ^13^C-NMR and mass spectrometric characterizations are described in the Supplementary Information.

### Photophysics of DA1 in solution

**Figure 2:**
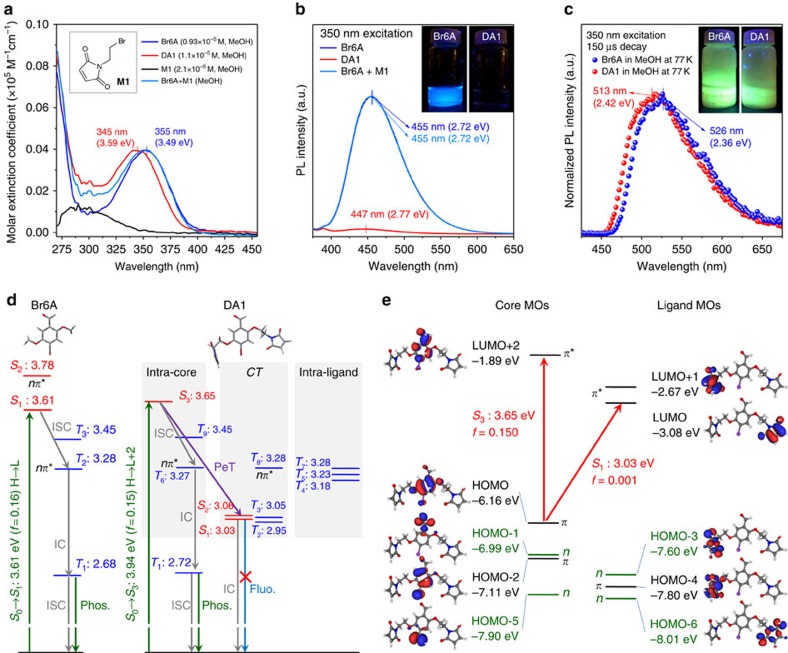
Photophysical properties of DA1 in a methanol solution. (**a**) Ultraviolet–visible absorption spectra of Br6A (0.93 × 10^−5^ M; blue line), DA1 (1.1 × 10^−5^ M; red line), M1 (2.1 × 10^−5^ M; black line) and Br6A/M1 (0.93 × 10^−5^ M/2.1 × 10^−5^ M; green line) in a methanol solution. Calculated ultraviolet–visible spectra of Br6A and DA1 using time-dependent density functional theory (TD-DFT) are shown in Supplementary Fig. S1, which are in good qualitative agreement with the experimental data. Inset shows the chemical structure of M1. (**b**) Photoluminescence (PL) spectra of methanol solutions of Br6A, DA1 and Br6A/M1 excited at *λ*_ex_=350 nm. The inset shows PL images of methanol solutions of Br6A and DA1 at RT under a 365-nm hand-held ultraviolet light. (**c**) Gated PL spectra of methanol solutions of Br6A and DA1 excited at *λ*_ex_=350 nm at 77 K. The inset shows PL images of methanol solutions of Br6A and DA1 at 77 K under a 365-nm hand-held ultraviolet light. (**d**) Term diagrams of Br6A and DA1 in methanol as calculated by TD-DFT; nπ*-type states are indicated, whereas the other states are all of ππ* character. Energies are given in eV. (**e**) MO diagram of DA1, as calculated at the DFT (B3LYP//BHLYP/6-311G*) level of theory.

We first investigated the solution-state luminescent properties of DA1 in methanol, showing distinctively different photophysics from Br6A. Although Br6A is fluorescent with a quantum yield of *Φ*_F_=12% at *λ*_max_=455 nm, DA1 shows negligible fluorescence at room temperature (RT) and ambient air pressure (Fig. 2b). However, at 77 K, both compounds show noticeable phosphorescence (Fig. 2c). Time-dependent density functional theory was used to understand the difference between Br6A and DA1.

According to the calculations, electronic excitation to the first excited singlet state (*S*_1_) of Br6A in methanol is mainly attributed to a HOMO→LUMO (ππ*) transition (Fig. 2d and Supplementary Table 1), calculated at 3.61 eV (343 nm) with considerable oscillator strength (*f*=0.16), from which Br6A can efficiently fluoresce. Alternatively, ISC from *S*_1_ to *T*_*n*_ can occur. This process is promoted by the heavy atom effect of bromine and by the energetically close nature of the *T*_2_ state. As the T_2_ state is nπ*, ISC from *S*_1_(ππ*) to *T*_2_(nπ*) is allowed by the El-Sayed rule and is followed by rapid relaxation to the *T*_1_ state (Fig. 2d). At ambient conditions, *T*_1_ effectively deactivates non-radiatively by ISC and consecutive thermal relaxation to *S*_0_, as well as by quenching by oxygen, enabled by effective diffusion in solution during the long *T*_1_ lifetime of Br6A. Lowering the temperature to 77 K restricts the ISC pathway and oxygen diffusion in the frozen environment, allowing for Br6A phosphorescence.

In DA1, the HOMO is slightly stabilized due to the presence of the maleimide groups on the side chains, so that the energetically lowest core-centred singlet state of ππ* nature (*S*_3_; Fig. 2e and Supplementary Table 1), which dominates the absorption spectrum, is blue shifted by 0.04 eV compared with the corresponding state of Br6A (that is, *S*_1_; Fig. 2d), in a qualitative agreement with the experimental data (0.10 eV; Fig. 2a). In contrast to Br6A, an effective quenching pathway for fluorescence is operative in DA1 due to the presence of the maleimide ligands. In fact, the lowest unoccupied maleimide MOs are lower in energy compared with the core-centred one, so that electronic excitation from the core-centred HOMO leads to nearly degenerated excited singlet states of *S*_1_ and *S*_2_ with complete charge-transfer (CT) character (Fig. 2d). Photoinduced electron transfer (PeT) from the originally excited, bright Franck–Condon state (*S*_3_) to the optically dark CT state (*S*_1_) effectively quenches the fluorescence of DA1. In any case, the ISC pathway to the (intra-core centred) triplet manifold in DA1 is identical to that in Br6A (Fig. 2d), so that at low temperature phosphorescence is noticeable in both systems, just slightly blue shifted in DA1 in agreement with experimental data (Fig. 2c).

### Characterization of Diels-Alder reaction

We prepared a DA1-doped PFMA film for characterizing the Diels–Alder reaction. The synthesized polymer, PFMA (1.0 wt%), was dissolved in chloroform (CHCl_3_) and mixed with the phosphor DA1 (1.2 wt% of DA1 for PFMA). The mixed solution was drop-cast on a pre-cleaned glass substrate and kept at RT for 10 min. The resulting drop-cast film was thermally annealed at 120 °C for 20 min under nitrogen atmosphere, leading to the formation of covalent bonding between DA1 and PFMA. For comparison, a Br6A-doped PFMA film (1.0 wt% of Br6A for PFMA) was also prepared in the same way.

**Figure 3:**
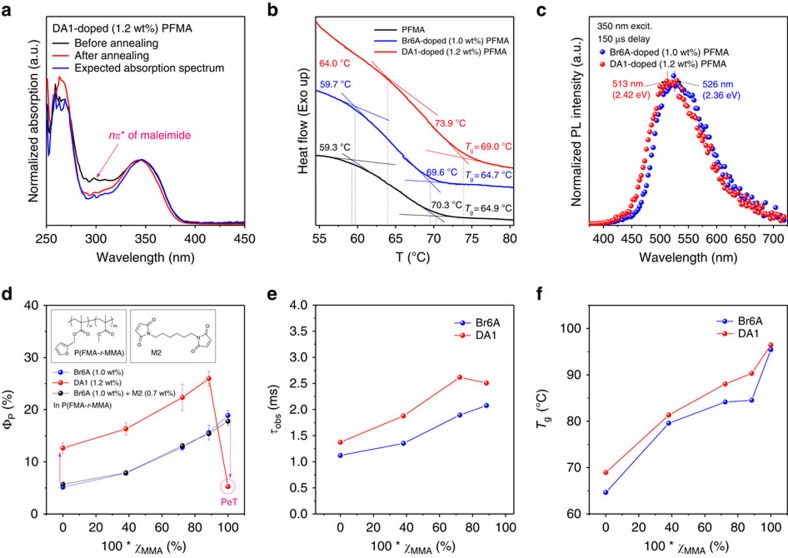
Photophysical and thermal characterization of Br6A- and DA1-doped polymer blend films. (**a**) Ultraviolet–visible absorption spectra of DA1-doped PFMA (1.2 wt% of DA1 for PFMA polymer) films before (black line) and after (red line) thermal annealing. Blue line shows the expected absorption spectrum of DA1-doped PFMA. (**b**) Differential scanning calorimetry (DSC) curves of Br6A- and DA1-doped PFMA (blue and red line, respectively) and PFMA (black line). (**c**) Gated PL spectra of Br6A- and DA1-doped PFMA at RT. (**d**) Phosphorescence quantum yield (*Φ*_P_) of Br6A- (blue line), DA1-doped (red line), Br6A and M2-doped (black line) P(FMA-*r*-MMA) at different *χ*_MMA_. Chemical structures of P(FMA-*r*-MMA) and M2 are given in inset. (**e**) Phosphorescence lifetime (*τ*_P_) and (**f**) glass transition temperature (*T*_g_) of Br6A- (blue line) and DA1-doped (red line) P(FMA-*r*-MMA) at different *χ*_MMA_.

To characterize the Diels–Alder reaction in the resulting polymer blend film, ultraviolet–visible spectroscopy was employed, which is a standard quantitative analysis tool commonly used for this purpose^30^. The maleimide moiety, M1, exhibited a typical absorption peak at around 300 nm, as seen in the M1 spectrum in Fig. 2a, which corresponds to an nπ* transition of the conjugated diene as confirmed by time-dependent density functional theory calculations (see Supplementary Fig. 1). Although in the sample before thermal annealing the M1 band is present, it disappeared after thermal annealing due to the Diels–Alder reaction (Fig. 3a). The degree of conversion of the Diels–Alder reaction can be estimated by calculating the expected DA1 spectrum after thermal annealing by subtracting the M1 spectrum from the DA1 spectrum before thermal annealing (for details on the procedure, see Supplementary Fig. 2); from this, a conversion efficiency of >95% can be estimated. Differential scanning calorimetry further confirmed the success of the Diels–Alder reaction, showing a notable increase of the glass transition temperature (*T*_g_) of the annealed DA1-doped PFMA compared with the unreacted pristine PFMA or Br6A-doped PFMA (Fig. 3b). This is attributed to the fact that cross-linking via the Diels–Alder reaction limits the mobility of polymer chains, resulting in an increase of *T*_g_. Finally, the success of the cross-linking reaction can be seen in the rise of fluorescence in the annealed DA1-doped PFMA film, that is, compared with the DA1-doped PMMA film and solution, which demonstrates the shutdown of the PeT process via the CT state by the Diels-Alder reaction.

### Phosphorescence properties of the polymer blend film

To identify the cross-linking effect on the phosphorescence properties, gated photoluminescence spectra, phosphorescence quantum yield (*Φ*_P_) and phosphorescence lifetime (*τ*_P_) of DA1- and Br6A-doped polymer films were measured at RT (Fig. 3c–e and Table 1). To minimize oxygen effects on the phosphorescence processes, all processes for sample preparation from drop-casting to packaging with an epoxy-seal were done under a nitrogen atmosphere (in a controlled atmosphere). To avoid aggregation-induced quenching, we optimized the doping concentration of phosphors (1.2 wt% of DA1 and 1.0 wt% of Br6A for polymers, respectively) through the measurement of *Φ*_P_ of phosphor-doped polymer films at different doping concentration of phosphors (Supplementary Fig. 3). It should be noted that DA1-doped polymer films were thermally annealed at 120 °C for 20 min, to induce cross-linking between DA1 and polymers. For comparison, Br6A-doped polymer films were prepared in exactly the same way as the DA1-doped polymer films. Additional details for sample preparation and measurement procedure are described in the Supplementary Information.

The resulting Br6A- and DA1-doped PFMA films showed noticeable phosphorescence at 526 nm (2.36 eV) and 513 nm (2.42 eV) with lifetimes of 2.0 and 2.6 ms, respectively (Fig. 3c,e). The observed blue shift of 0.06 eV in the emission spectrum of DA1-doped PFMA film is due to the presence of maleimide moieties at the side chain, corresponding well to that in methanol solution as mentioned in the section ‘Photophysics of DA1 in solution'. The rigid environment of the polymer matrix and exclusion of oxygen allow for noticeable RTP in both Br6A- and DA1-doped PFMA films, in contrast to those in methanol solution. Notably, *Φ*_P_ was measured to be 13% for the DA1-doped PFMA film, which is *ca*. 2.5 times larger than that of the Br6A-doped PFMA film having no covalent linkages (*Φ*_P_=5%; Fig. 3d). As discussed in more detail below, we attribute this large enhancement in *Φ*_P_ to the fact that covalent linkages restrict the molecular motions in the proximity of embedded phosphors and hence effectively suppress the radiationless relaxation pathways.

We first investigated the effect of molecular weight of phosphors on the emission efficiency to confirm that the enhancement of *Φ*_P_ purely originates from the formation of cross-linking. To study the effect of molecular weight, we prepared DA1 analogues, DA2 and DA3, through side-chain modification of DA1, while maintaining the core structure of DA1 (Supplementary Fig. 4). Newly synthesized DA2 and DA3 with the extended length of alkyl chain have a larger molecular weight than DA1. As shown in Supplementary Fig. 4, in the cross-linked systems phosphorescence quantum efficiency decreases with increasing molecular weight. On the other hands, DA1 showed much higher efficiency than that of Br6A, although DA1 has a larger molecular weight than Br6A. Thus, we could conclude that molecular weight does not affect the phosphorescence efficiency.

The rigidity of glassy polymers is usually represented by *T*_g_ (ref. 31). We therefore further investigated the effect of *T*_g_ of the polymer matrix on the phosphorescence of phosphor-doped polymer films by synthesizing ene-reactive random copolymers, P(FMA-*r*-MMA), comprising FMA and MMA with various mole ratios, allowing for specific control of *T*_g_ of the synthesized copolymers (Fig. 3d–f and Supplementary Fig. 5). *T*_g_ are higher in copolymers with a larger MMA mole ratio (*χ*_MMA_=number of MMA/number of MMA and FMA) due to the fact that the relatively short and stiff structure of MMA limits the mobility of polymer chains^32^. Figure 3d,e summarize the measured *Φ*_P_ and *τ*_P_ of Br6A- and DA1-doped P(FMA-*r*-MMA) films with various *χ*_MMA_. As the *χ*_MMA_increases, the measured *Φ*_P_ and *τ*_P_ increase in both Br6A- and DA1-doped P(FMA-*r*-MMA) films, corresponding to a decrease of the non-radiative decay, consistent with the increase of *T*_g_. Remarkably, the measured *Φ*_P_ of DA1-doped P(FMA-*r*-MMA) films is *ca*. two times larger than that of Br6A-doped P(FMA-*r*-MMA) films at all *χ*_MMA_, which correlates well with the increase of *T*_g_ in that range (it is noteworthy that the exceptionally low *Φ*_P_ of DA1-doped PMMA film arises from the activation of PeT process by the maleimide moieity; see section ‘Photophysics of DA1 in solution'). It should be stressed that *Φ*_P_ of the DA1-doped P(FMA-*r*-MMA) film for *χ*_MMA_=0.88 reaches *ca*. 28%, which is comparable to *Φ*_P_ of crystals of phosphorescent materials reported in the literature^10^,^14^,^15^,^16^. However, the increased *T*_g_ cannot fully explain the enhancement of *Φ*_P_, because *Φ*_P_ of the DA1-doped P(FMA-*r*-MMA) films for *χ*_MMA_=0.72 and 0.88 is higher than that of the Br6A-doped PMMA film having higher *T*_g_ (Fig. 3d,f), indicating that the molecular motions in the vicinity of phosphors are of high importance.

To examine our hypothesis, we measured the *Φ*_P_ of DA1-doped P(FMA-*r*-MMA) with different doping concentration of DA1. As DA1 successfully forms cross-links with polymer matrix through Diels–Alder click reaction, we can control the extent of cross-links by changing the doping concentration of DA1. Br6A-doped P(FMA-*r*-MMA) films were prepared for comparison. As shown in Supplementary Fig. 3, the phosphorescence quantum efficiency decreases with increasing doping concentration of DA1. The aggregation-induced emission quenching by increased doping concentration may affect dominantly the emission efficiency (based on the results of Br6A, we clearly conclude that aggregation-induced quenching is the main reason for the reduction of phosphorescence quantum yield) and further increase of cross-links (the increase of matrix rigidity) merely affect the phosphorescence efficiency. This result also indicates that rigidity in the proximity of phosphors is much more important than the matrix rigidity represented by the glass transition temperature.

To further confirm our argument, we additionally measured *Φ*_P_ of Br6A (1.0 wt% of Br6A for polymers) and M2 (0.72 wt% of M2 for polymers)-doped P(FMA-*r*-MMA) films (Fig. 3d). M2 only acts as a cross-linker, as the core structure of DA1 is not incorporated in M2. As shown in Fig. 3d, Br6A and M2-doped P(FMA-*r*-MMA) films showed almost the same value of *Φ*_P_ with Br6A-doped P(FMA-*r*-MMA) films at all *χ*_MMA_, which is much smaller than DA1-doped P(FMA-*r*-MMA). Therefore, we clearly conclude that the restricted molecular motions in the vicinity of phosphors by cross-linking between phosphors and polymer matrix is the most important factor to suppress the radiationless decay and hence, enhance the phosphorescence efficiency.

### Excited state kinetics analysis

**Figure 4:**
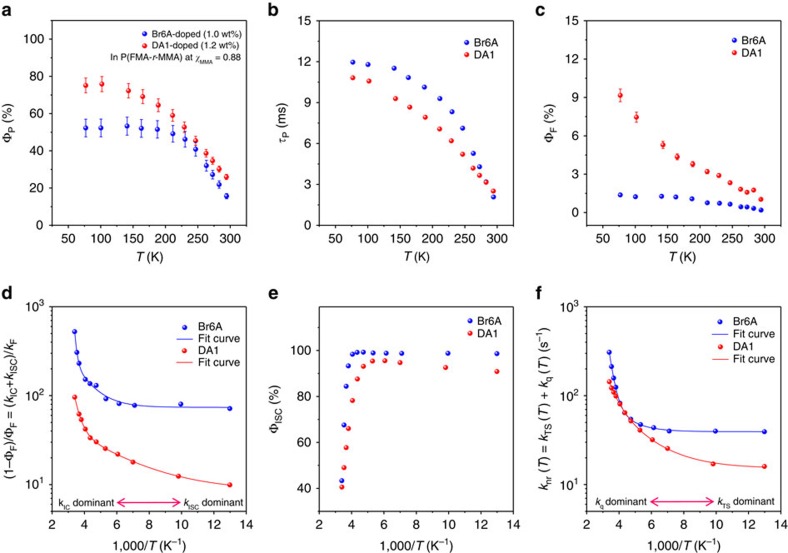
Temperature-dependent measurements of photophysical properties of Br6A- and DA1-doped P(FMA-*r*-MMA) blend films. Temperature-dependent plots of Br6A- (blue dots) and DA1-doped (red dots) P(FMA-*r*-MMA) blend films at *χ*_MMA_=0.88 for (**a**) *Φ*_P_, (**b**) *τ*_P_, (**c**) fluorescence quantum yield (*Φ*_F_) and (**e**) ISC quantum yield (*Φ*_ISC_) are shown. (**d**) Temperature-dependent plots of Br6A- (blue dots) and DA1-doped (red dots) P(FMA-*r*-MMA) blend films at *χ*_MMA_= 0.88 for (1–*Φ*_F_)/Φ_F_ are shown. The fitted curves (blue and red lines, respectively) by a bi-exponential decay model are shown together. (**f**) The non-radiative decay rates (*k*_nr_) of Br6A- (blue dots) and DA1-doped (red dots) films calculated from equations (2) and (3) are plotted versus 1,000/*T*. The fitted curves (blue and red lines, respectively) by a bi-exponential decay model are shown together.

To fully understand the underlying principles of *Φ*_P_ enhancement of DA1-doped PFMA and P(FMA-*r*-MMA) films, we performed temperature (*T*)-dependent measurements of the photoluminescent properties for Br6A- and DA1-doped P(FMA-*r*-MMA) films of *χ*_MMA_=0.88, as shown in Fig. 4a–c. We analysed the characteristics of the photophysical processes from the measured photophysical values of *Φ*_P_, *τ*_P_ and *Φ*_F_, as a function of *T*. To obtain the quantum efficiency for ISC from *S*_1_ to *T*_*n*_ (*Φ*_ISC_), the value of (1–*Φ*_F_)/*Φ*_F_ for the Br6A- and DA1-doped P(FMA-*r*-MMA) films is plotted as a function of *T* (Fig. 4d). The resulting plots can be fitted well by a bi-exponential decay model, allowing for the extraction of the ratio of the rate constants for IC (*k*_IC_) and ISC from *S*_1_ to *T*_*n*_ (*k*_ISC_). Then, *Φ*_ISC_ can be determined at a given temperature based on equation (1) (Fig. 4e; the detailed procedure for extracting the ratio *k*_IC_/*k*_ISC_ by curve fitting and deriving the equation (1) are given in the Supplementary Information).


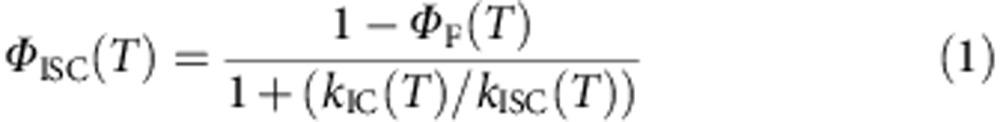


Having *Φ*_ISC_ at hand, the rate constants for phosphorescence process (*k*_P_) and non-radiative deactivation processes (*k*_nr_) are then obtained through equations (2) and (3) (Fig. 4f and Table 1).


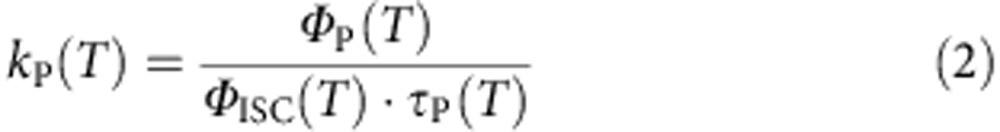



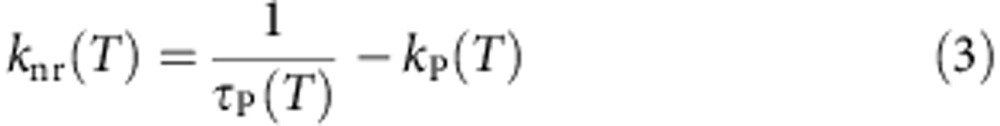


Interestingly, the calculated non-radiative deactivation of the triplet at RT is much smaller in the DA1-doped P(FMA-*r*-MMA) film (*k*_nr_=143.5 s^−1^) compared with the Br6A-doped P(FMA-*r*-MMA) film (*k*_nr_=308.0 s^−1^), indicating that the enhancement of Φ_P_ by cross-linking is mainly due to the suppression of the radiationless relaxation pathways (Table 1). Although the reduced non-radiative rate is the major contributor to the phosphorescence enhancement, contribution of an increased radiative rate is also non-negligible (Table 1; the contribution of radiative and non-radiative processes on the phosphorescence enhancement is calculated in the Supplementary Information). The reason for the increase in *k*_P_ cannot be found in the nature of *T*_1_, which is suggested to be very similar in energy (both from experiment and calculations), nor in the electronic composition (CI and MO topology). Thus, the most probable reason for the difference is the screening by the local environment, as in a first approximation the radiative rate should scale with *n*^2^ (with *n* being the refractive index), which should be higher in DA1 due to the presence of polar maleimide groups.

**Figure 5:**
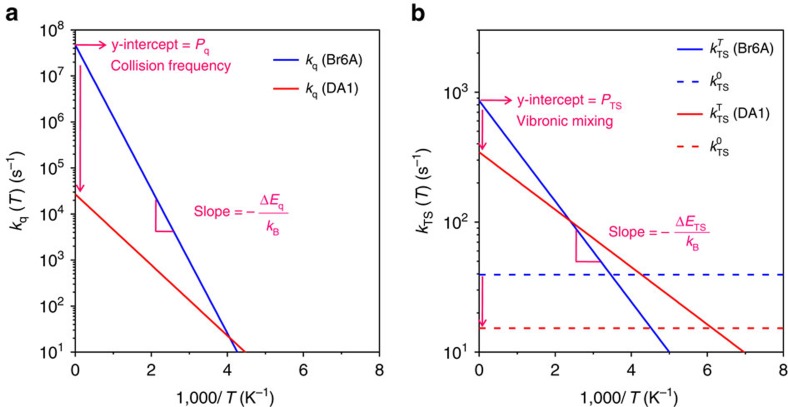
The Arrhenius plots of *k*_TS_ and *k*_q_ versus 1,000/T. (**a**) *k*_q_ and (**b**) *k*_TS_^T^ (solid lines) and *k*_TS_^0^ (dotted lines), extracted by fitting curves of *k*_nr_ versus *T* (Fig. 4f), are plotted versus 1,000/*T*. Pre-exponential factors (*P*_q_ and *P*_TS_) and activation energy (Δ*E*_q_ and Δ*E*_TS_) of Dexter-type triplet ET process (left) and ISC from *T*_1_ to *S*_0_ (right) were determined by equations (5) and (6), respectively.

To analyse the radiationless deactivation processes in more detail, the non-radiative decay for the Br6A- and DA1-doped P(FMA-*r*-MMA) films are plotted as a function of *T* (Fig. 4f), which displays a clear bi-exponential decay, allowing for the extraction of the rate constant for ISC from *T*_1_ to *S*_0_ (*k*_TS_) and the rate constant for the quenching processes of triplet state of an embedded phosphor by interaction with the host matrix and/or oxygen (*k*_q_) based on equations (4, 5, 6) (Fig. 5a,b).


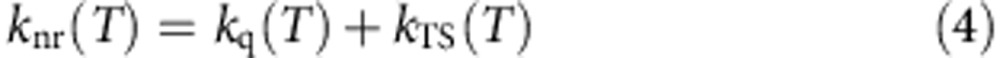



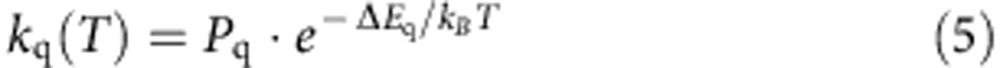



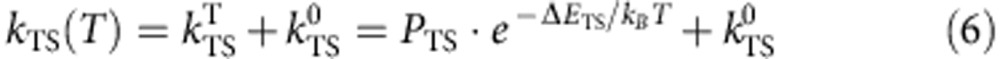


where *k*_TS_^T^ is the rate constant for *T*-dependent ISC from *T*_1_ to *S*_0_, *k*_TS_^0^ is the rate constant for *T*-independent ISC from *T*_1_ to *S*_0_, *P*_q_ and Δ*E*_q_ are the pre-exponential factor and activation energy of the quenching processes, respectively, *P*_TS_ and Δ*E*_TS_ are the pre-exponential factor and activation energy of *T*-dependent ISC from *T*_1_ to *S*_0_, respectively, and *k*_B_ is the Boltzmann constant. As detailed below, *k*_TS_ contributes mainly to the radiationless processes at lower temperatures, whereas *k*_q_ dominates at higher temperatures.

At RT, *k*_q_ might include quenching processes by endothermic triplet–triplet ET from phosphors to the host matrix^21^,^23^, through aggregation formation, or by the presence of oxygen^2^. However, in this study we could minimize the quenching processes by oxygen and aggregation, because the experiments were conducted under inert atmosphere and at low doping concentration (*ca*. 1 wt% of phosphors for copolymers). Thus, *k*_q_ should be caused by Dexter-type ET from *T*_1_ of the phosphors to triplet states of the polymer matrix and to small amount of residual oxygen (<0.1 p.p.m.) via collision between the reacting partners. Notably, the evaluated *k*_q_ at RT is greatly reduced in the DA1-doped P(FMA-*r*-MMA) film (*k*_q_=65.7 s^−1^), being 3.4 times smaller than that of the Br6A-doped P(FMA-*r*-MMA) film (*k*_q_=223.5 s^−1^; Fig. 5a and Table 1). The large reduction of *k*_q_(*RT*) is mainly attributed to a decrease in the pre-exponential factor (*P*_q_; Fig. 5a and Table 1) by three orders of magnitude, as evaluated from Arrhenius plots of *k*_q_ versus *T* based on equation (5), which represents the collision frequency according to diffusion-controlled triplet ET theory^33^. We could clearly conclude that the restriction of diffusion/translational motion of phosphors and of polymer chains effectively suppresses the Dexter-type triplet ET process by minimizing the number of collisions between reacting partners and hence significantly increases *Φ*_P_.

The activation energies of Dexter-type triplet ET (Δ*E*_q_) can be determined from the slope of the Arrhenius plot of equation (5) (*k*_q_ versus *T*; Fig. 5a and Table 1). However, the calculated activation energies (0.311 and 0.152 eV for Br6A and DA1, respectively) are much smaller than the difference between the *T*_1_ energy of the phosphors (*ca*. 2.36 and 2.42 eV for Br6A and DA1, respectively) and *T*_1_ energy of the polymer matrices (*ca*. 3.1 and 3.85 eV for PMMA^22^ and 2-methylfuran^34^, respectively). We thus hypothesize that quenching may occur via a dark CT state formed between the phosphors and the carbonyl groups of polymers, as previously described in literature^35^.

Interestingly, the pre-exponential factor (*P*_TS_) of the DA1-doped P(FMA-*r*-MMA) film evaluated from the Arrhenius plots of *k*_TS_ versus *T* based on equation (6) is *ca*. 2.5 times smaller than that of the Br6A-doped P(FMA-*r*-MMA) film (Fig. 5b and Table 1). The *T*-independent ISC process (*k*_TS_^0^) is also significantly suppressed in the DA1-doped P(FMA-*r*-MMA) film (Fig. 5b and Table 1). We believe that the restriction of specific vibrational motions (for example, out-of-plane vibrational modes, *vide infra*) of the phosphor that are effective in mixing of zero-order electronic states of *T*_1_ and *S*_0_ (that is, vibronic mixing through Herzberg–Teller vibronic coupling and/or diabatic (non-Born–Oppenheimer) coupling caused by the nuclear momentum^36^,^37^,^38^,^39^), by cross-linking, may be attributed to the decrease in *P*_TS_ and *k*_TS_^0^. In fact, recent quantum chemical calculations of a related molecule (that is, psoralen) indicate that out-of-plane vibration modes allow for efficient ISC from a ππ*-type singlet to a ππ*-type triplet, driven by Herzberg–Teller vibronic coupling, which is in good support of our argument^38^. Despite the meaningful reduction in *P*_TS_ and *k*_TS_^0^, *k*_TS_(*RT*) did not significantly change due to an unexpected decrease of Δ*E*_TS_ in the DA1-doped P(FMA-*r*-MMA) film. Further optimized material development through elaborate engineering of Δ*E*_TS_ is required to additionally enhance *Φ*_P_.

### Phosphorescent properties in a variety of copolymers

**Figure 6:**
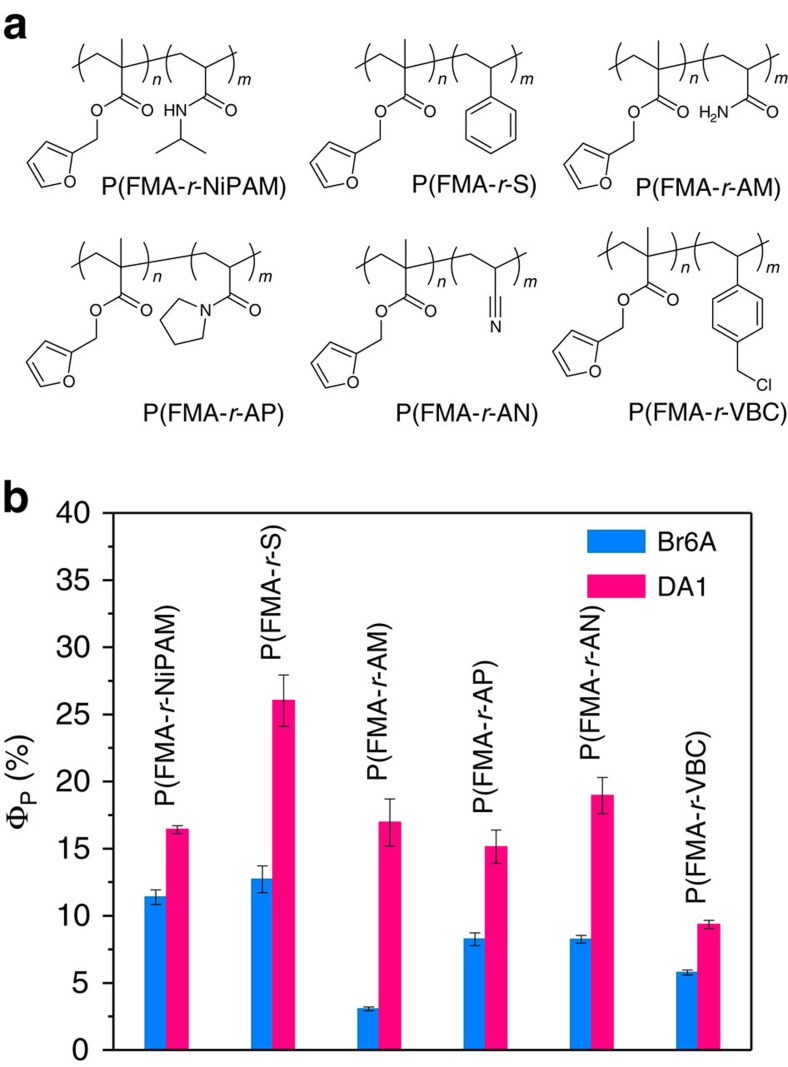
Phosphorescence QY of various Br6A- and DA1-doped copolymers. (**a**) Chemical structures of various copolymers are shown. (**b**) Phosphorescence QY of various Br6A- (sky blue) and DA1-doped (pink) copolymers are presented. DA1-doped copolymers show higher phosphorescence QY than those of Br6A-doped copolymers.

To test the applicability of our approach, we designed a series of copolymers containing a variety of monomers (Fig. 6a). A full description of the syntheses and characterizations of designed copolymers is described in the Supplementary Information. As anticipated, DA1-doped copolymers all exhibited significantly enhanced *Φ*_P_ compared with Br6A-doped copolymers (Fig. 6b), indicating that our method can be generally applied for enhanced RTP of metal-free organic materials.

## Discussion

In summary, we have developed a new design rule for highly efficient RTP in metal-free organic materials. Covalent linking between phosphors and a polymer matrix not only provides convenient access to enhanced RTP of metal-free organic materials in a variety of polymer matrices but also enables better fundamental understanding of the influence of molecular motions on the radiationless relaxation pathways. Our experimental and theoretical study might allow for the development of next-generation organic phosphorescent materials that may give new impetus to the field of organic optoelectronics, bio-imaging, sensing and data security.

## Methods

### Preparation of Br6A- and DA1-doped polymer films for photophysical measurements at RT

PMMA, PFMA, P(FMA-*r*-MMA), P(FMA-*r*-S), P(FMA-*r*-NiPAM), P(FMA-*r*-AM), P(FMA-*r*-AP), P(FMA-*r*-AN) and P(FMA-*r*-VBC) (1 wt%) were dissolved in chloroform (CHCl_3_) and mixed with the phosphor (1.0 wt% of Br6A and 1.2 wt% of DA1 for polymers, respectively). The mixed solutions were drop-cast on a pre-cleaned glass substrate and kept at RT for 10 min. The resulting drop-cast films were thermally annealed at 120 °C for 20 min and kept in a vacuum chamber for 30 min, to completely remove residual solvent and oxygen. The resulting polymer films were packaged by attaching a glass lid to the polymer films using an epoxy seal (EPOXY TECHNOLOGY 305) around the perimeter and kept at RT for 24 h. All processes from drop-casting to packaging were done in a nitrogen-filled glove box.

### Preparation of Br6A- and DA1-doped polymer films for temperature-dependent measurements

P(FMA-*r*-MMA) (1 wt%) with *χ*_MMA_=0.88 was dissolved in chloroform (CHCl_3_) and mixed with the phosphor (1.0 wt% of Br6A and 1.2 wt% of DA1 for polymers, respectively). The mixed solutions were drop-cast on sapphire substrates and kept at RT for 10 min. The resulting drop-cast films were thermally annealed at 120 °C for 20 min and kept in a vacuum chamber for 30 min, to remove residual solvent and oxygen.

## Additional information

**How to cite this article:** Kwon, M. S. *et al.* Suppressing molecular motions for enhanced room-temperature phosphorescence of metal-free organic materials. *Nat. Commun.* 6:8947 doi: 10.1038/ncomms9947 (2015).

## Supplementary Material

Supplementary Information Supplementary Figures 1-7, Supplementary Tables 1-4, Supplementary Methods and Supplementary References

## Figures and Tables

**Table 1:** Photophysical data of Br6A- and DA1-doped P(FMA-*r*-MMA) blend films for *χ*_MMA_=0.88 at RT.

**System**	***Φ***_**P**_**/%**	***τ***_**P**_**/ms**	**Φ**_**ISC**_**/%**	***k***_**P**_**/s**^−1^	***k***_**nr**_**/s**^−1^	**k**_**q**_**/s**^−1^	***k*_TS_^T^/s**^−1^	***k*_TS_^0^/s**^−1^	**Δ*****E***_**q**_**/**_**eV**_	***P***_**q**_**/**	**Δ*****E***_**TS**_**/eV**	***P***_**TS**_**/**
Br6A	15.6	2.08	43.3	173.5	308.0	223.5	41.5	39.4	0.311	4.74 × 10^7^	0.077	8.59 × 10^2^
DA1	26.0	2.51	40.6	255.1	143.5	65.7	61.2	15.2	0.152	269 × 10^4^	0.044	3.45 × 10^2^

DA1, diene-reactive phosphor; *k*_q_, rate contant for quenching processes; *k*_nr_, rate contant for nonradiative decay*; k*_P_, rate constants for phosphorescence; RT, room temperature;

Measured *Φ*_P_, *τ*_P_ and *Φ*_ISC_, and the calculated *k*_P_, *k*_nr_, *k*_q_ and temperature-(in)dependent intersystem crossing from *T*_1_ to *S*_0_ (*k*_TS_^T^ and *k*_TS_^0^). Activation energies (Δ*E*_TS_ and Δ*E*_q_) and pre-exponential factors (*P*_TS_ and *P*_q_) are extracted from Arrhenius plots of *k*_q_ versus *T* and *k*_TS_ versus *T* based on equations (5) and (6), respectively.
