# Supplementary material for: Suppressing molecular motions for enhanced room-temperature phosphorescence of metal-free organic materials
Source: Nat Commun. 2015 Dec 2;6:8947. doi: 10.1038/ncomms9947 (PMC4686823; doi:10.1038/ncomms9947)
Supplement: Supplementary Information — Supplementary Figures 1-7, Supplementary Tables 1-4, Supplementary Methods and Supplementary References [file ncomms9947-s1.pdf]

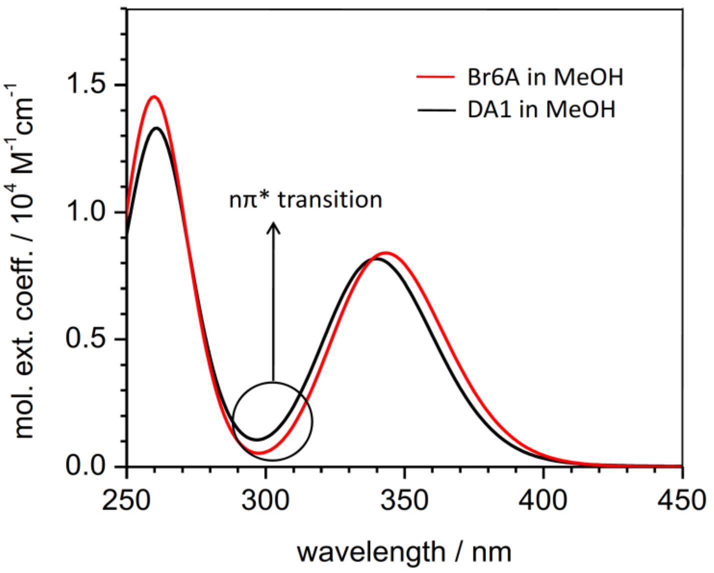

Supplementary Figure 1. Calculated UV-Vis spectra of methanol solutions of Br6A and DA1 from TD-DFT. The absorption peak at 307 nm corresponds to an nπ* transition from the aldehyde to the maleimide moiety, consistent with the experimental spectrum shown in Fig. 1a and Fig. 2a.

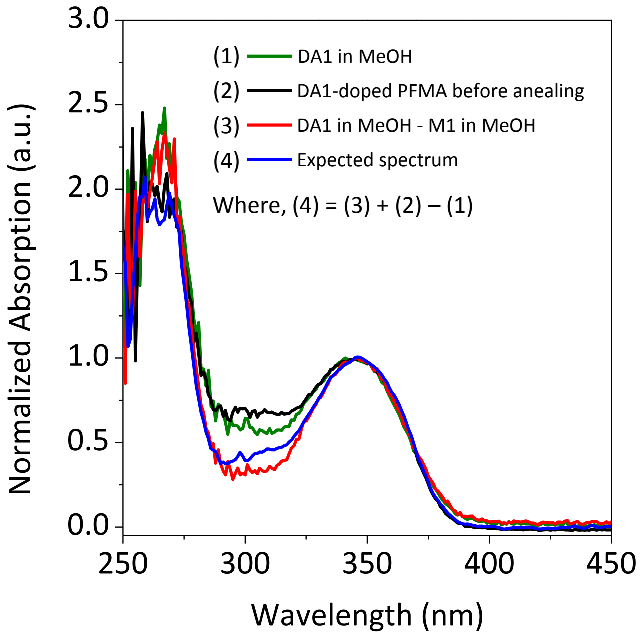

Supplementary Figure 2. Calculation of expected UV-Vis absorption spectrum of DA1-doped PFMA.

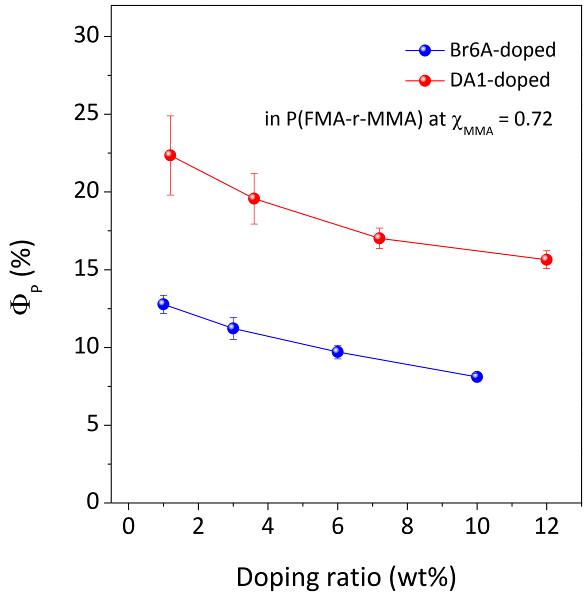

Supplementary Figure 3. Phosphorescence quantum yield (Φ_P) of Br6A- and DA1-doped P(FMA-*r*-MMA) at x_{MMA}=0.72 with different doping concentration of phosphors.

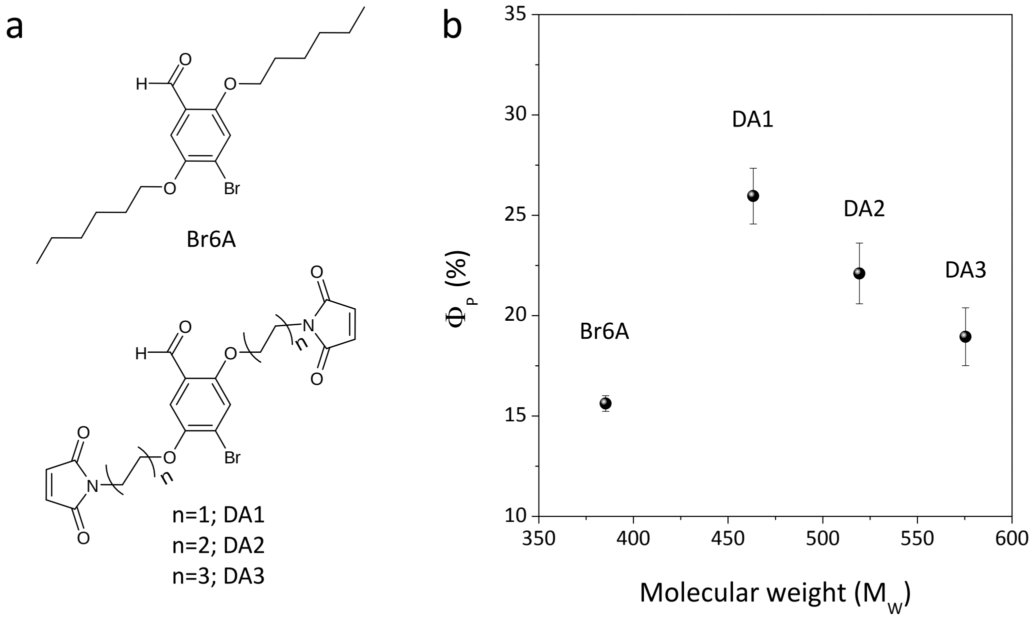

Supplementary Figure 4. (a) Chemical structures of Br6A, DA1, DA2, and DA3 are given. (b) Phosphorescence quantum yield (Φ_P) of Br6A-, DA1-, DA2-, and DA3-doped P(FMA-*r*-MMA) at x_{MMA}=0.88.

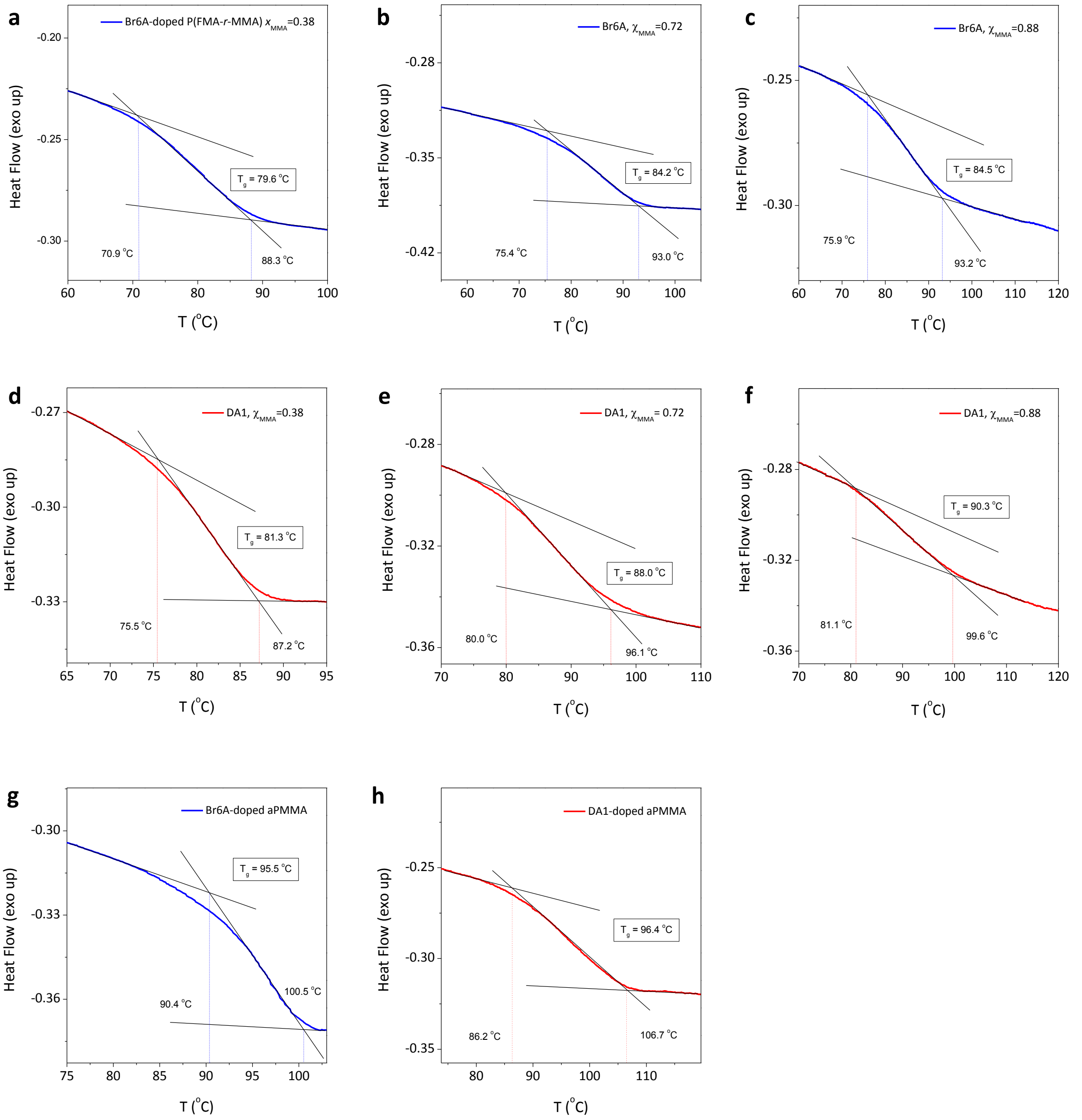

Supplementary Figure 5. Differential scanning calorimetry (DSC) curves of Br6A- and DA1- doped P(FMA-*r*-MMA) at different x_{MMA}.

a

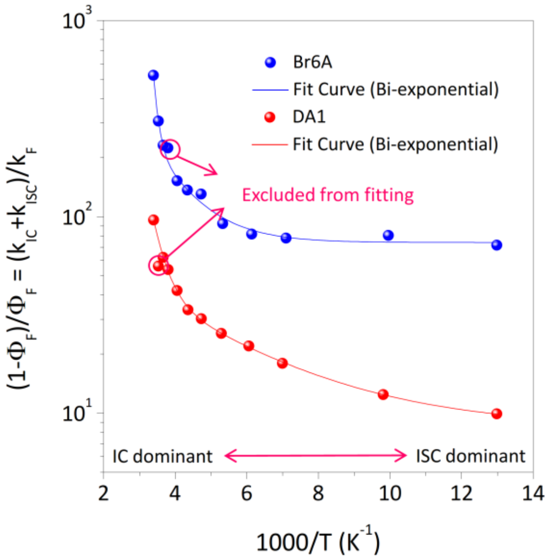

b

	A_{IC}	C_{IC}	A_{ISC}	C_{ISC}	y_{ISC}^0	*Adj.* R^2
Br6A	1.73x10^{14}	0.125	3.73x10^{3}	1.06	7.39x10^{1}	0.997
DA1	1.18x10^{6}	0.342	9.19x10^{1}	3.12	8.46	0.999

Supplementary Figure 6. Determination of intersystem crossing quantum yield (Φ_{ISC}). (a) Temperature-dependent plots of Br6A- (blue dots) and DA1-doped (red dots) P(FMA-*r*-MMA) blend films at χ_{MMA} = 0.88 for $(1-\Phi_F)/\Phi_F$ are shown. (b) The results of curve fitting are presented. The detailed procedure for extracting the ratio (k_{IC}/k_{ISC}) by curve fitting and deriving Equation 1 are given in Supplementary Methods.

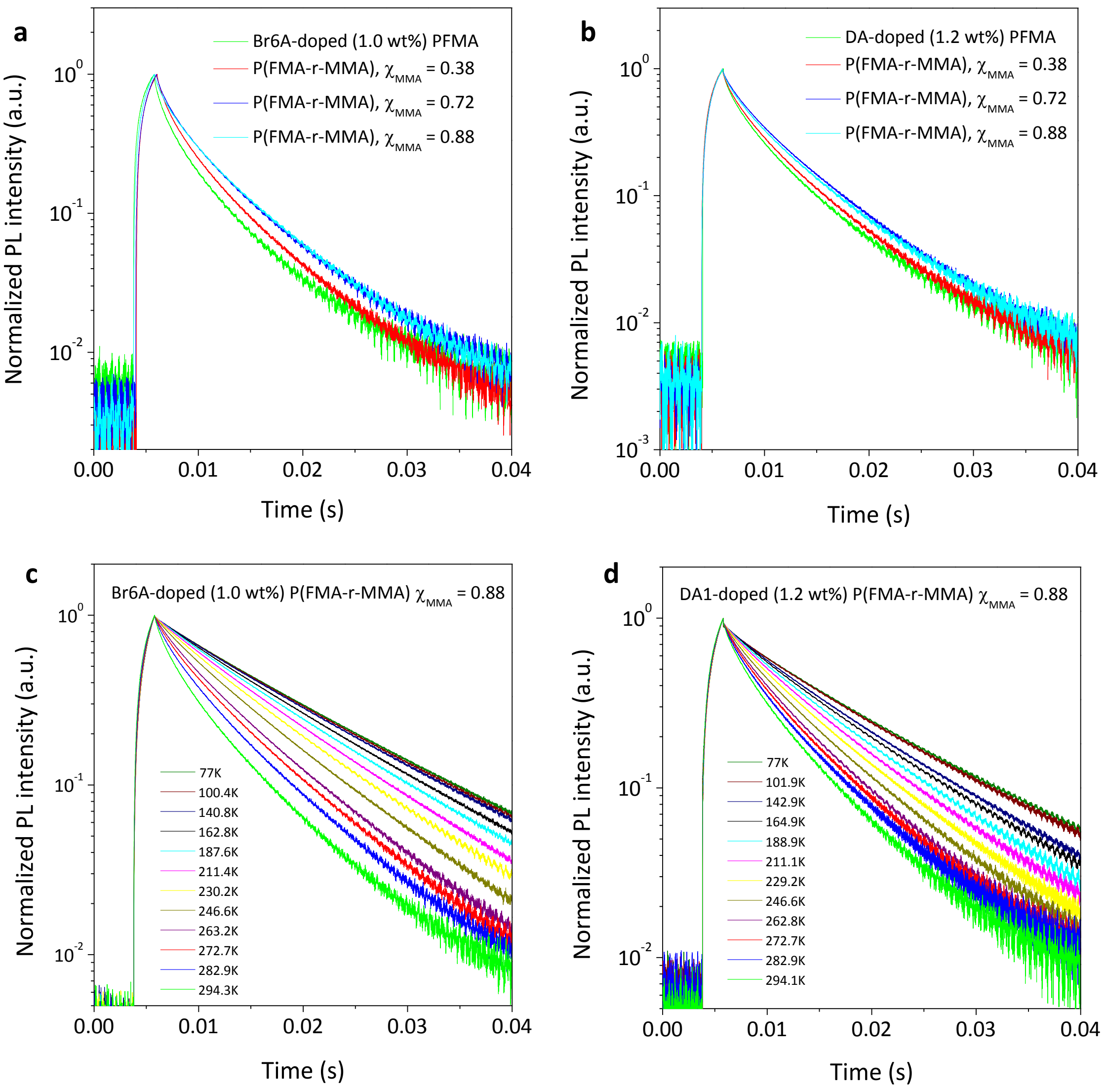

Supplementary Figure 7. The obtained phosphorescence profiles for (a) Br6A-doped (1.0 wt%) P(FMA-*r*-MMA), and (b) DA1-doped (1.2 wt%) P(FMA-*r*-MMA) at different x_{MMA}, and (c) Br6A-doped (1.0 wt%) P(FMA-r-MMA) and (d) DA1-doped (1.2 wt%) P(FMA-r-MMA) at x_{MMA} =0.88 at different temperatures

Supplementary Table 1. TD-DFT calculated singlet and triplet states of Br6A and DA1: energy (E), oscillator strength (*f*), configuration interaction (CI) description (H = HOMO, L= LUMO); only contributions >10% are listed.

solvent	state	E / eV (λ / nm)	*f*	main CI configuration	localization	nature
Br6A	T_1	2.68 (463)		H→L (96%)		ππ*
	T_2	3.28 (378)		H-2→L (93%)		nπ*
	T_3	3.45 (359)		H-1→L (33%)		ππ*
	S_1	3.61 (343)	0.16	H→L (96%)		ππ*
	S_2	3.78 (328)	0.00	H-2→L (96%)		nπ*
DA1	T_1	2.72 (455)		H→L+2 (94%)	core	ππ*
	T_2	2.95 (420)		H→L (79%)<br>H-6→L (10%)	CT	ππ*
	T_3	3.05 (407)		H→L+1 (92%)<br>H-4→L+1 (5%)	CT	ππ*
	T_4	3.18 (390)		H-4→L+1 (47%)<br>H-11→L+1 (29%)<br>H→L+1 (6%)<br>H-6→L+1 (6%)	ligand	ππ*
	T_5	3.23 (384)		H-6→L (31%)<br>H-12→L (28%)<br>H→L (19%)<br>H-9→L (7%)	ligand	nπ*
	T_6	3.27 (379)		H-2→L+2 (73%)<br>H-1→L+2 (18%)	core	ππ*
	T_7	3.28 (378)		H-3→L+1 (94%)	ligand	ππ*
	T_8	3.28 (378)		H-5→L (92%)	CT	nπ*
	T_9	3.45 (360)		H-1→L+2 (55%)<br>H-2→L+2 (15%)<br>H→L+4 (11%)	core	nπ*
	S_1	3.03 (410)	0.001	H→L (99%)	CT	ππ*
	S_2	3.06 (364)	0.000	H→L+1 (100%)	CT	ππ*
	S_3	3.65 (340)	0.150	H→L+2 (95%)	core	ππ*

Supplementary Table 2. The results of fitting of phosphorescence profiles for Br6A- and DA1-doped P(FMA-*r*-MMA) at different x_{MMA} at RT.

	x_{MMA}	a_1	τ_1 / (ms)	a_2	τ_2 / (ms)	τ_{obs} /	χ^2
Br6A	0.88	1.57	1.27	0.0689	5.98	2.08	1.40E-8
	0.72	2.38	1.26	0.0838	5.80	1.89	1.54E-8
	0.38	5.57	1.09	0.0776	5.26	1.35	1.27E-8
	PFMA	6.52	0.964	0.0555	4.80	1.12	1.14E-8
DA1	0.88	0.848	1.40	0.0631	5.99	2.51	1.15E-8
	0.72	1.57	1.44	0.123	6.13	2.62	2.18E-8
	0.38	2.31	1.26	0.0853	5.62	1.88	1.54E-8
	PFMA	3.99	1.07	0.0648	5.21	1.37	1.27E-8

Supplementary Table 3. The results of fitting of phosphorescence profiles for Br6A- and DA1-doped P(FMA-*r*-MMA) at x_{MMA} =0.88 at different temperatures.

	T / (K)	a_1	τ_1 / (ms)	a_2	τ_2 / (ms)	τ_{obs} /	χ^2
Br6A	77	0.0233	3.80	0.0774	12.7	12.0	3.30E-9
(x_{MMA}=0.88)	100.4	0.0243	3.79	0.0795	12.5	11.8	3.65E-9
	140.8	0.0257	3.78	0.0827	12.3	11.5	4.67E-9
	162.8	0.0300	3.76	0.0976	11.6	10.8	3.35E-9
	187.6	0.0364	3.68	0.0901	10.9	10.1	4.83E-9
	211.4	0.0496	3.38	0.105	10.2	9.29	3.54E-9
	230.2	0.0741	2.90	0.110	9.44	8.32	8.78E-9
	246.6	0.126	2.53	0.110	8.66	7.12	5.21E-9
	263.2	0.299	1.98	0.101	7.75	5.27	9.16E-9
	272.7	0.475	1.73	0.0950	7.32	4.29	1.22E-8
	282.9	0.806	1.50	0.0849	6.74	3.18	1.42E-8
	294.3	1.57	1.27	0.0689	5.98	2.08	1.40E-8
DA1	77	0.0359	3.22	0.0646	12.0	10.8	9.36E-9
(x_{MMA}=0.88)	101.9	0.0377	3.25	0.0670	11.7	10.6	1.16E-8
	142.9	0.0536	2.92	0.0764	10.5	9.29	6.07E-9
	164.9	0.0626	2.77	0.0774	9.99	8.67	8.07E-9
	188.9	0.0799	2.62	0.0801	9.40	7.93	1.03E-8
	211.1	0.110	2.39	0.0823	8.76	7.06	8.50E-9
	229.2	0.151	2.18	0.0827	8.16	6.19	8.45E-9
	246.6	0.224	1.98	0.0805	7.56	5.21	7.51E-9
	262.8	0.351	1.76	0.0763	7.00	4.19	1.04E-8
	272.7	0.453	1.64	0.0730	6.72	3.66	1.44E-8
	282.9	0.573	1.55	0.0683	6.41	3.16	1.79E-8
	294.1	0.848	1.40	0.0631	5.99	2.51	1.15E-8

Supplementary Table 4. Characterization of newly synthesized polymers

Entry	Reaction time (h)	Feed ratio		Composition		M_n / (g/mol)	PDI
		FMA	Co-monomer	FMA	Co-monomer		
PFMA		10	0	1	0	145,400	2.24
P(FMA-*r*-MMA)		7	3	0.618	0.382	142,700	2.01
P(FMA-*r*-MMA)		3	7	0.277	0.723	136,700	2.16
P(FMA-*r*-MMA)		1	9	0.116	0.884	138,900	2.04
P(FMA-*r*-S)		1	9	0.127	0.873	34,300	1.72
P(FMA-*r*-NiPAM)		1	9	0.116	0.884	41,600	1.99
P(FMA-*r*-AM)		1	9	0.295	0.705	.	.
P(FMA-*r*-AP)		1	9	0.268	0.732	138,500	8.87
P(FMA-*r*-AN)		1	9	0.313	0.687	.	.
P(FMA-*r*-VBC)		1	9	0.293	0.707	39,800	7.71

Supplementary Methods

1. Sample preparations

1.1. Chemicals

Unless otherwise specified, all chemicals were purchased commercially, and used without further purification. Azobisisobutyronitrile (AIBN) was purified by recrystallization from methanol. Poly (methyl methacrylate) (PMMA) (Aldrich, M_w = 120,000 g/mol) was used without further purification. Br6A and M2 were synthesized following previously reported synthetic routes.[1,2] Poly(furfuryl methacrylate) (PFMA), Poly(furfuryl methacrylate-*r*-methyl methacrylate) P(FMA-*r*-MMA), Poly(furfuryl methacrylate-*r*-styrene) P(FMA-*r*-S), Poly(furfuryl methacrylate-*r*-*N*-isopropylacrylamide) P(FMA-*r*-NiPAM), Poly(furfuryl methacrylate-*r*-acryloyl pyrrolidine) P(FMA-*r*-AP), Poly(furfuryl methacrylate-*r*-acrylonitrile) P(FMA-*r*-AN), Poly(furfuryl methacrylate-*r*-4-vinylbenzyl chloride) P (FMA-*r*-VBC), DA1, DA2, and DA3 were synthesized such as following.

1.1.1. Synthesis of polymers

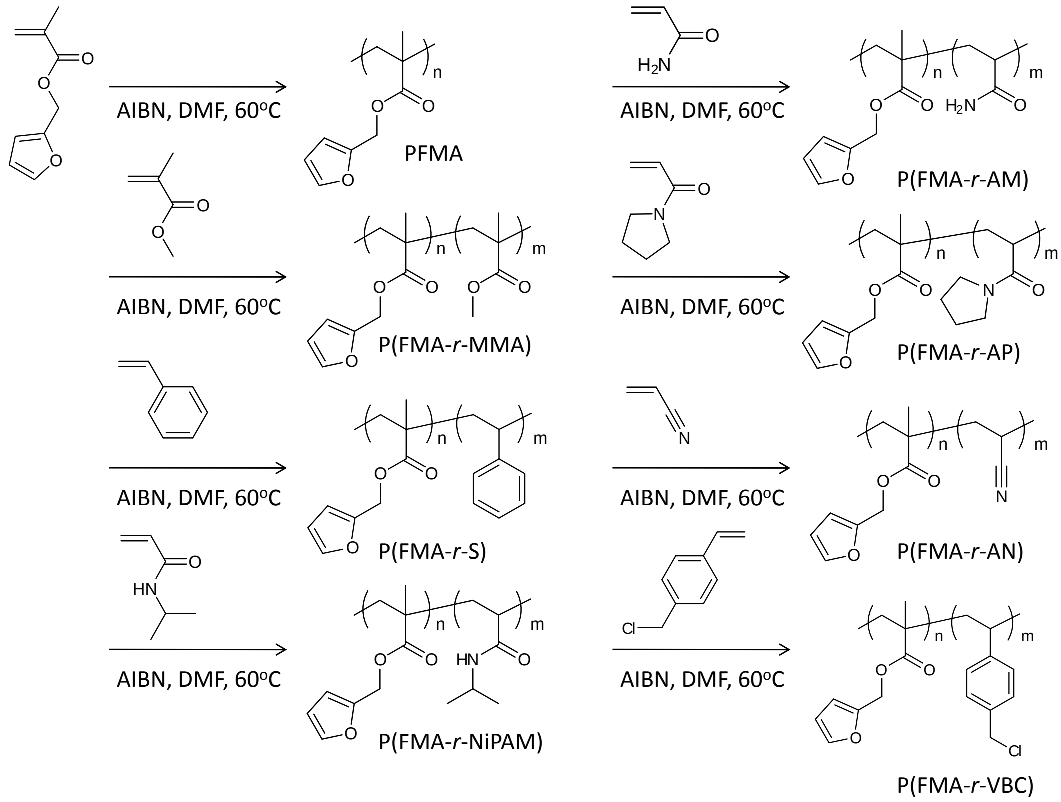

General procedure for polymerization

Furfuryl methacrylate (FMA) and co-monomer were polymerized via free radical polymerization using AIBN as the initiator. In the polymerization process, furfuryl methacrylate, and the co-monomer at the specified feed ratio (see Supplementary Table 4), and AIBN were dissolved in DMF, and the solution was purged with argon for 30 min. The monomers/solvent weight ratio was kept at ~30% and the reaction was carried out at 60 °C for desired time. The resulting copolymers were precipitated in diethyl ether or methanol and dried under vacuum overnight.

1.1.2. Synthesis of DA1, DA2, and DA3

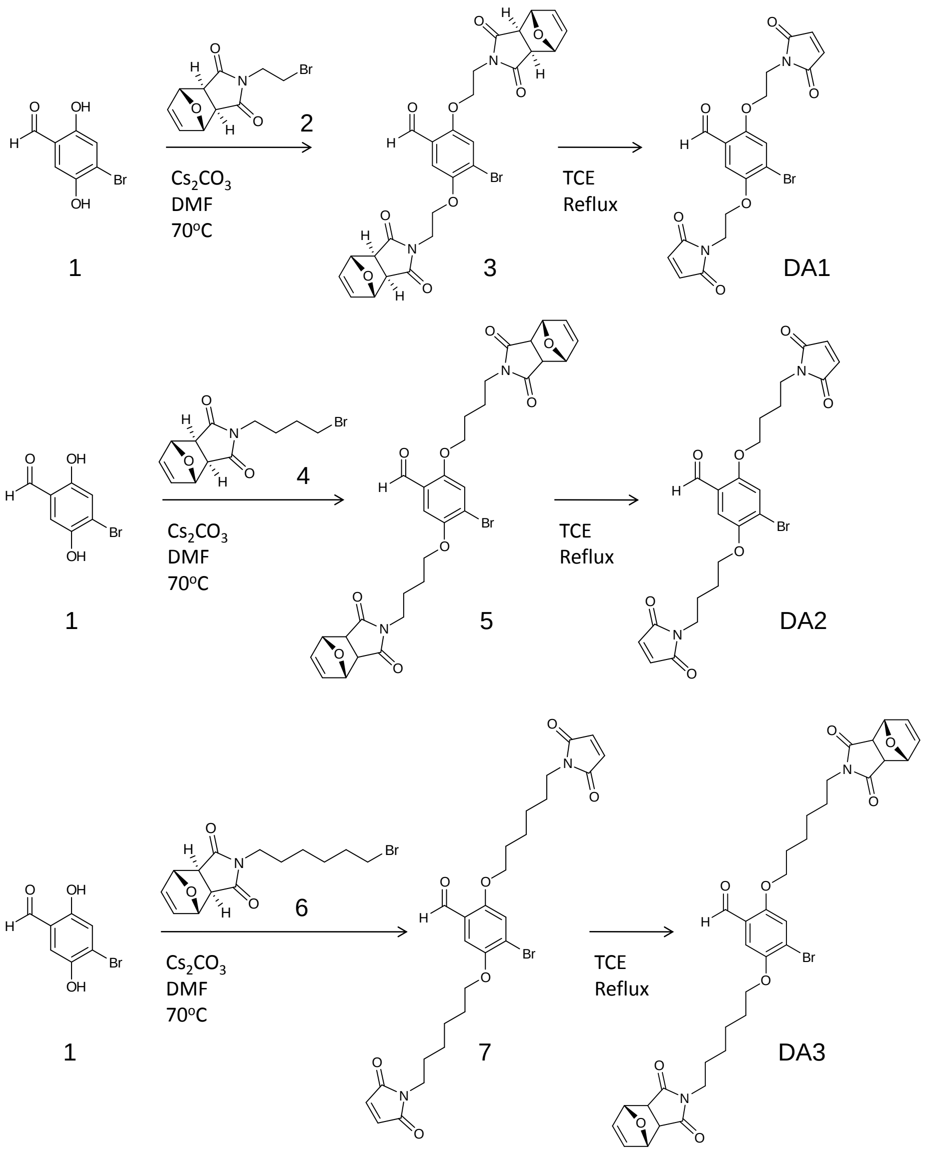

Synthesis and characterization of DA1, DA2, and DA3

DA1. To a solution of **1** (205 mg, 0.94 mmol) and **2** (566 mg, 2.08 mmol) in DMF (9.4 ml) was added Cs_2CO_3 (677 mg, 2.08 mmol) at RT. The solution was stirred at 70 ˚C for 10 h. After cooling to room temperature, the mixture was poured into 1 M HCl and extracted with ethyl acetate and washed with water and brine. The organic phase was dried over $MgSO_4$ and the solvent was evaporated *in vacuo*. Purification of the residue by flash column chromatography (hexanes-acetone, 1:1) gave 3 as the major product with an inseparable, unidentified mixture.

Tetrachloroethatne (TCE, 10 ml) solution of the resulting product mixture was heated under reflux for overnight. After cooling to room temperature, the mixture was purified by flash column chromatography (hexanes-acetone, 4:1) and gave DA1 as an off-white solid powder (67 mg, 15% for 2 steps). ^{1}H NMR (400 MHz, $CDCl_3$): δ 10.24 (s, 1 H), 7.26 (s, 1 H), 7.13 (s, 1 H), 6.72 (s, 4H), 4.16 (t, 4H, J = 5.4 Hz), 3.97 (t, 4H, J = 5.4 Hz). ^{13}C NMR (100 MHz, $CDCl_3$): δ 188.7, 170.6, 170.5, 155.2, 149.7, 134.5, 134.5, 124.5, 121.0, 118.3, 111.3, 66.3, 66.2, 37.1, 37.0. MS *m*/*z* (ESI+, relative intensity): 948 (33), 487 (48), 463 (M^++1, 99), 284 (9), 256 (12), 130 (24), 124 (11). HRMS (ESI+) calcd. for $C_{19}H_{16}BrN_2O_7$ (M^++1) 463.0141, found 463.0135.

DA2. To a solution of **1** (55 mg, 0.253 mmol) and **4** (190 mg, 0.633 mmol) in DMF (2.5 ml) was added Cs_2CO_3 (206 mg, 0.633 mmol) at RT. The solution was stirred at 70 ˚C for 10 h. After cooling to room temperature, the mixture was poured into 1 M HCl and extracted with ethyl acetate and washed with water and brine. The organic phase was dried over $MgSO_4$ and the solvent was evaporated *in vacuo*. Purification of the residue by flash column chromatography (hexanes-acetone, 1:1) gave **5** as the major product with an inseparable, unidentified mixture.

Tetrachloroethatne (TCE, 3 ml) solution of the resulting product mixture was heated under reflux for overnight. After cooling to room temperature, the mixture was purified by flash column chromatography (hexanes-acetone, 6:1) and gave DA2 as an off-white solid powder (58 mg, 44% for 2 steps). ^{1}H NMR (400 MHz, $CDCl_3$): δ 10.38 (s, 1 H), 7.29 (s, 1 H), 7.21 (s, 1 H), 6.71 (s, 4H), 4.07−4.01 (m, 4H), 3.64−3.58 (m, 4H), 1.83−1.81 (m, 8H). ^{13}C NMR (100 MHz, $CDCl_3$): δ 188.8, 171.0, 155.7, 150.0, 134.4, 134.3, 124.5, 121.2, 118.7, 111.0, 69.2, 68.8, 37.7, 37.5, 26.6, 26.5, 25.5,

25.4. MS *m*/*z* (ESI+, relative intensity): 543 (57), 519 (M^{+}+1, 87), 443 (22), 359 (54), 283 (40), 256 (12), 152 (29), 122 (30). HRMS (ESI+) calcd. for $C_{23}H_{24}BrN_2O_7$ (M^{+}+1) 519.0767, found 519.0761.

DA3. To a solution of **1** (350 mg, 1.61 mmol) and **6** (1.11 g, 3.38 mmol) in DMF (16 ml) was added Cs_2CO_3 (1.10 g, 3.38 mmol) at RT. The solution was stirred at 70 ˚C for 10 h. After cooling to room temperature, the mixture was poured into 1 M HCl and extracted with ethyl acetate and washed with water and brine. The organic phase was dried over $MgSO_4$ and the solvent was evaporated *in vacuo*. Purification of the residue by flash column chromatography (hexanes-acetone, 1:1) gave **7** as the major product with an inseparable, unidentified mixture.

Tetrachloroethatne (TCE, 20 ml) solution of the resulting product mixture was heated under reflux for overnight. After cooling to room temperature, the mixture was purified by flash column chromatography (hexanes-acetone, 6:1) and gave DA3 as an off-white solid powder (348 mg, 38% for 2 steps). ^{1}H NMR (400 MHz, $CDCl_3$): δ 10.39 (s, 1 H), 7.29 (s, 1 H), 7.21 (s, 1 H), 6.69 (s, 4H), 4.01 (t, 4H, *J* = 5.4 Hz), 3.53 (t, 4H, *J* = 5.4 Hz), 1.84−1.64 (m, 4H), 1.63−1.59 (m, 4H), 1.55−1.48 (m, 4H), 1.40−1.34 (m, 4H). ^{13}C NMR (100 MHz, $CDCl_3$): δ 189.1, 171.1, 155.9, 150.0, 134.3, 122.5, 121.2, 118.6, 110.9, 69.8, 69.4, 38.0, 37.9, 29.2, 29.1, 28.7, 28.6, 26.7, 26.6, 25.8, 25.7. MS *m*/*z* (ESI+, relative intensity): 575 (M^{+}+1, 95), 413 (21), 359 (36), 283 (19), 177 (7). HRMS (ESI+) calcd. for $C_{27}H_{32}BrN_2O_7$ (M^{+}+1) 575.1393, found 575.1387.

1.2. Synthetic details

The progress of reactions were checked on TLC plates (Merck 5554 Kiesel gel 60 F254), and the spots were visualized under 254 nm (and/or 365 nm) UV light and/or charring after dipping the TLC plate into a vanillin solution (9.0 g of vanillin and 1.5 mL of concentrated sulfuric acid in 300 mL of MeOH) or $KMnO_4$ solution (3 g of $KMnO_4$, 20 g of K_2CO_3, and 5 mL of 5% NaOH solution in 300 mL of water). Column chromatography was performed on silica gel (Merck 9385 Kiesel gel 60). Unless otherwise specified, all reactions were conducted under a slight positive pressure of dry nitrogen. The usual work-up refers to washing the quenched reaction mixture with brine, drying the combined organic extracts over anhydrous $MgSO_4$ and evaporating under reduced pressure using a

rotary evaporator.

1.3. Preparation of Br6A- and DA1-doped polymer blend films for photophysical measurements at RT

1 wt% of PMMA, PFMA, P(FMA-*r*-MMA), P(FMA-*r*-S), P(FMA-*r*-NiPAM), P(FMA-*r*-AM), P(FMA-*r*-AP), P(FMA-*r*-AN), and P(FMA-r-VBC) were dissolved in chloroform ($CHCl_3$) and mixed with the phosphor (1.0 wt% of Br6A and 1.2 wt% of DA1 for polymers, respectively). The mixed solutions were drop-cast on a pre-cleaned glass substrate and kept at room temperature (RT) for 10 min. The resulting drop-cast films were thermally annealed at 120 ^{o}C for 20 min and kept in a vacuum chamber for 30 min to completely remove residual solvent and oxygen. The resulting polymer films were packaged by attaching a glass lid to the polymer films using an epoxy seal (EPOXY TECHNOLOGY 305) around the perimeter and kept at RT for 24 h. All processes from drop-casting to packaging were done in a nitrogen filled glove box.

2. Sample measurements

2.1. Characterization of synthesized molecules and polymers

Newly synthesized small molecules were characterized by ^{1}H-NMR, ^{13}C-NMR, and mass spectrometry. ^{1}H-NMR spectrum was recorded on a Varian, MR 400 (400 MHz) in $CDCl_3$ or DMSO-d_6 solution. ^{13}C-NMR spectrum was recorded on a Varian, MR400 (400 MHz) in $CDCl_3$ solution. Chemical shift values were recorded as parts per million relative to tetramethylsilane as an internal standard, and coupling constants in Hertz. Mass spectra were recorded on an Agilent Q-TOF 6520 system using electrospray ionization in positive ion detection (ESI+) mode. Significant fragments are reported in the following fashion: *m*/*z* (relative intensity).

The molecular weights (MWs) and MW distribution of polymers synthesized were determined by gel permeation chromatography (GPC, Water 1515, Isocratic HPLC pump) coupled with a refractive index (RI) detector (Waters 2414, RI detector) and three columns (Styragel HR 2, Styragel HR 3, Styragel HR 4, Waters). Chloroform was used as the eluent at 25˚C at a flow rate of 1.0 mL/min. Polysytrene standards were used for calibration. The polymer composition was determined using a

^{1}H-NMR spectrometer (Varian, MR 400 (400 MHz)) with $CDCl_3$ or DMSO-d_6 as the solvent.

2.2. Glass transition characterization.

Glass transition temperatures of polymers were determined by DSC using a TA Instruments Discovery Series DSC under nitrogen. Drop-cast polymer films were prepared using the same procedures described in section 1.3. Each dried film was ground into a fine powder and stored in a vacuum chamber for 2 h, after which it was sealed in a Tzero hermetic pan and used for DSC measurements. DSC samples were first heated to 150 ˚C at a rate of 10 ˚C min^{-1}, then cooled down to 20 ˚C at a rate of 10 ˚C min^{-1}, and finally heated at a rate of 10 ˚C min^{-1}; during this final heating, glass transition data were obtained.

2.3. Measurements of photophysical properties of solutions and films at RT

UV-visible absorption spectra were measured on a Varian Cary50 UV/Vis spectrophotometer. Photoluminescence and absolute phosphorescence quantum yield (Φ_P) were obtained using a Photon Technologies International (PTI) QuantaMaster spectrofluorometer equipped with an integrating sphere. Absorption and emission inside the sphere were determined by comparison to a blank sample (glass only). A neutral density filter was used to allow for maximization of the emission signal without saturating the photomultiplier tube detector with excitation light. Each sample type was run in quadruplicate with each quantum yield measurement coming from a freshly prepared sample that was sealed with epoxy resin under nitrogen atmosphere (detailed procedures for thin film preparation and sealing are described above). Measurements proved highly repeatable, and errors are given as ±1 standard deviation.

2.4. Phosphorescence lifetime (τ_P) measurement of polymer blend films

Transient lifetime measurements were taken using a silicon avalanche photodiode, (Hamamatsu, C12703) and oscilloscope (Tektronix DPO2024). The excitation source was a 365 nm (peak) LED (Mightex, MLS-0365) driven by a pulse generator (HP 4118A) with a pulse width of 2 ms. Mean

decay times τ_P were obtained from the individual lifetimes τ_i and amplitudes a_i of multi-exponential evaluation through Supplementary Equation 1.

$$\tau_P = \frac{\sum_i a_i \tau_i^2}{\sum_i a_i \tau_i} \qquad \text{(Supplementary Equation 1)}$$

The obtained phosphorescence profiles for all samples used in the experiments are shown in Supplementary Figure 7.The results of fitting of all samples are presented in Supplementary Table 2 and 3.

2.5. Temperature dependent measurement of photophysical properties of Br6A- and DA1-doped P(FMA-r-MMA) at x_{MMA} = 0.88

1 wt% of P(FMA-*r*-MMA) at x_{MMA} = 0.88 was dissolved in chloroform ($CHCl_3$) and mixed with the phosphor (1.0 wt% of Br6A and 1.2 wt% of DA1 for polymers, respectively). The mixed solutions were drop-cast on sapphire substrates and kept at RT for 10 mins. The resulting drop-cast films were thermally annealed at 120 ˚C for 20 mins and kept in a vacuum chamber for 30 mins to completely remove residual solvent and oxygen. Temperature dependent lifetime and PLQY measurements were carried out in an open cycle liquid nitrogen cryostat under vacuum (under 10^{-3} torr). Spectral and relative emission intensity measurements were taken using a spectrometer. The temperature dependent PLQY was calculated from the room temperature absolute PLQY (Section 2.3.) and temperature dependent relative emission intensity.

2.6. The detailed procedure for extracting k_{IC}/k_{ISC} by curve fitting

If we assumed that k_F is independent of temperature, the curves are fitted by a bi-exponential fitting model as shown in Supplementary Equation 2. This assumption is commonly reasonable since the fluorescence process has no activation energy, and hence it is independent of temperature, to a first approximation.

$$y = A_{IC} \cdot e^{-\frac{x}{C_{IC}}} + A_{ISC} \cdot e^{-\frac{x}{C_{ISC}}} + y^0_{ISC} \qquad \text{(Supplementary Equation 2)}$$

Where, $x = \frac{1000}{T}$ and $y = \frac{k_{IC} + k_{ISC}}{k_F} = y_{IC} + y_{ISC}$

The intersystem crossing (ISC) process is the main contributor at lower temperatures, whereas the internal conversion (IC) process dominates at higher temperatures. Hence,

$$y_{IC} = A_{IC} \cdot e^{-\frac{x}{C_{IC}}} \quad \text{(Supplementary Equation 3)}$$

$$y_{ISC} = A_{ISC} \cdot e^{-\frac{x}{C_{ISC}}} + y_{ISC}^{0} \quad \text{(Supplementary Equation 4)}$$

Where, $C_{IC} < C_{ISC}$. Thus, $\frac{k_{IC}}{k_{ISC}}$ is expressed by Supplementary Equation 5.

$$\frac{k_{IC}}{k_{ISC}} = \frac{(k_{IC} / k_F)}{(k_{ISC} / k_F)} = \frac{y_{IC}}{y_{ISC}} = \frac{A_{IC} \cdot e^{-\frac{x}{C_{IC}}}}{A_{ISC} \cdot e^{-\frac{x}{C_{ISC}}} + y_{ISC}^{0}} \quad \text{(Supplementary Equation 5)}$$

2.7. The detailed procedure for deriving Equation 1

Assume that our system does not have reverse intersystem crossing by thermally activated process and triplet-triplet annihilation process and quenching processes by energy transfer, through aggregation formation, or by chemical reaction. Then, by definition,

$$\Phi_F + \Phi_{ISC} + \Phi_{IC} = 1 \quad \text{(Supplementary Equation 6)}$$

From Equation S6,

$$\Phi_{IC} = 1 - \Phi_F - \Phi_{ISC} \quad \text{(Supplementary Equation 7)}$$

Dividing both sides by Φ_{ISC},

$$\frac{\Phi_{IC}}{\Phi_{ISC}} = \frac{1 - \Phi_F}{\Phi_{ISC}} - 1 \quad \text{(Supplementary Equation 8)}$$

Since $\Phi_F = k_F \cdot \tau_F$, $\Phi_{ISC} = k_{ISC} \cdot \tau_F$, and $\Phi_{IC} = k_{IC} \cdot \tau_F$, Supplementary Equation 8 can be expressed by Supplementary Equation 9.

$$\frac{k_{IC}}{k_{ISC}} = \frac{1 - \Phi_F}{\Phi_{ISC}} - 1 \quad \text{(Supplementary Equation 9)}$$

When Supplementary Equation 9 is transformed, Equation 1 can be obtained.

$$\Phi_{ISC} = \frac{1-\Phi_F}{1+\frac{k_{IC}}{k_{ISC}}} \qquad \text{(Equation 1)}$$

2.8. Calculation for the contribution of radiative and non-radiative decay

The efficiency of phosphorescence process (radiative decay from T_1 to S_0) can be expressed by Supplementary Equation 10.

$$\frac{\Phi_P}{\Phi_{ISC}} = \frac{k_P}{k_{nr}+k_P} = \frac{k_P}{\left(k_q + k_{TS}^{T} + k_{TS}^{0}\right)+k_P} \qquad \text{(Supplementary Equation 10)}$$

In Br6A-doped system,

$$\frac{\Phi_P(Br6A)}{\Phi_{ISC}(Br6A)} = \frac{k_P(Br6A)}{k_{nr}(Br6A)+k_P(Br6A)} = \frac{173.5}{(223.5+41.5+39.4)+173.5} = 0.36$$

If k_P increases without decreasing k_{nr},

$$\frac{\Phi_P(rad)}{\Phi_{ISC}(rad)} = \frac{k_P(rad)}{k_{nr}(rad)+k_P(rad)} = \frac{255.1}{(223.5+41.5+39.4)+255.1} = 0.453$$

If k_{nr} decreases without increasing k_p,

$$\frac{\Phi_P(nonrad)}{\Phi_{ISC}(nonrad)} = \frac{k_P(nonrad)}{k_{nr}(nonrad)+k_P(nonrad)} = \frac{173.5}{(65.7+61.2+15.2)+173.5} = 0.547$$

If k_{nr} decreases and k_p increase (DA1-doped system),

$$\frac{\Phi_P(DA1)}{\Phi_{ISC}(DA1)} = \frac{k_P(DA1)}{k_{nr}(DA1)+k_P(DA1)} = \frac{255.1}{(65.7+61.2+15.2)+255.1} = 0.639$$

Thus, contribution of radiative and non-radiative decay processes can be such as following:

$$Contribution(rad) = \frac{(0.453-0.36)}{(0.453-0.36)+(0.547-0.36)} \times 100(\%) = 33(\%)$$

$$Contribution(nonrad) = \frac{(0.547-0.36)}{(0.453-0.36)+(0.547-0.36)} \times 100(\%) = 67(\%)$$

3. Computational details

3.1. Quantum-chemical calculations

Geometry optimization (G.O.) was carried out at the density functional theory DFT level of theory. The BHandHLYP functional (6-311G* basis set; as implemented in the Gaussian09 program package.[3]) was chosen, providing reliable geometries.[4] This is of crucial importance in the present case, since the time-dependent (TD) calculations turned out to be very sensitive, even to small changes in the geometry.   Due to the flexible linkers connecting the maleimide ligands to the Br6A core, the potential surface of DA1 gave several stable minima (absence of negative frequencies); the most stable one being a structure with a practically coplanar structure of the alkoxy functionalities and the Br6A core, just like in Br6A itself. MO calculations as well as the time dependent (TD) part were done by employing the B3LYP functional (6-311G* basis set), being a reasonable compromise between accuracy and computational cost. Furthermore, relative energies of triplets and singlet (CT) states are reasonably well treated byB3LYP.[5] Solvent effects were taken into account by the implicit polarizable continuum model (PCM).

Supplementary References

1. O, Bolton, K. Lee, H. J. Kim, K. Y. Lin, J. Kim, *Nat. Chem.* **2011**, *3*, 205

2. McGrath, D. V. et al. *Tetrahedron Lett.* **2015**, 5011

3. Gaussian 09, Revision D.01, M. J. Frisch, G. W. Trucks, H. B. Schlegel, G. E. Scuseria, M. A. Robb, J. R. Cheeseman, G. Scalmani, V. Barone, B. Mennucci, G. A. Petersson, H. Nakatsuji, M. Caricato, X. Li, H. P. Hratchian, A. F. Izmaylov, J. Bloino, G. Zheng, J. L. Sonnenberg, M. Hada, M. Ehara, K. Toyota, R. Fukuda, J. Hasegawa, M. Ishida, T. Nakajima, Y. Honda, O. Kitao, H. Nakai, T. Vreven, J. A. Montgomery, Jr., J. E. Peralta, F. Ogliaro, M. Bearpark, J. J. Heyd, E. Brothers, K. N. Kudin, V. N. Staroverov, R. Kobayashi, J. Normand, K. Raghavachari, A. Rendell, J. C. Burant, S. S. Iyengar, J. Tomasi, M. Cossi, N. Rega, J. M. Millam, M. Klene, J. E. Knox, J. B. Cross, V. Bakken, C. Adamo, J. Jaramillo, R. Gomperts, R. E. Stratmann, O. Yazyev, A. J. Austin, R. Cammi, C. Pomelli, J. W.

Ochterski, R. L. Martin, K. Morokuma, V. G. Zakrzewski, G. A. Voth, P. Salvador, J. J. Dannenberg, S. Dapprich, A. D. Daniels, Ö. Farkas, J. B. Foresman, J. V. Ortiz, J. Cioslowski, and D. J. Fox, Gaussian, Inc., Wallingford CT, 2009.

4. (a) G. Macchi, B. Milián Medina, M. Zambianchi, R. Tubino, J. Cornil, G. Barbarella, J. Gierschner, F. Meinardi, *Phys. Chem. Chem. Phys.* **2009**, 11, 984; (b) B. Milián Medina, D. Wasserberg, S. C. J. Meskers, E. Mena-Osteritz, P. Bäuerle, J. Gierschner, *J. Phys. Chem. A* **2008**, 112, 13282

5. B. Milián-Medina, J. Gierschner, *Org. Electron.* **2012**, 13, 895
